# A Rabbit Model for Peripheral Nerve Reconstruction Studies Avoiding Automutilation Behavior

**DOI:** 10.1055/s-0042-1747959

**Published:** 2022-06-21

**Authors:** Jonathan A. Sorkin, Ziv Rechany, Mara Almog, Nina Dietzmeyer, Yuval Shapira, Kirsten Haastert-Talini, Shimon Rochkind

**Affiliations:** 1 Research Center for Nerve Reconstruction, Department of Neurosurgery, Tel Aviv Sourasky Medical Center, Tel Aviv, Israel; 2 Division of Peripheral Nerve Reconstruction, Department of Neurosurgery, Tel Aviv Sourasky Medical Center, Tel Aviv University, Tel Aviv, Israel; 3 Institute of Neuroanatomy and Cell Biology, Hannover Medical School, Hannover, Germany; 4 Center for Systems Neuroscience (ZSN), Hannover, Germany

**Keywords:** peripheral nerve reconstruction, rabbit model, automutilation behavior

## Abstract

**Background** The rabbit sciatic nerve injury model may represent a valuable alternative for critical gap distance seen in humans but often leads to automutilation. In this study, we modified the complete sciatic nerve injury model for avoiding autophagy.

**Materials and Methods** In 20 adult female New Zealand White rabbits, instead of transecting the complete sciatic nerve, we unilaterally transected the tibial portion and preserved the peroneal portion. Thereby loss of sensation in the dorsal aspect of the paw was avoided. The tibial portion was repaired in a reversed autograft approach in a length of 2.6 cm. In an alternative repair approach, a gap of 2.6 cm in length was repaired with a chitosan-based nerve guide.

**Results** During the 6-month follow-up period, there were no incidents of autotomy. Nerve regeneration of the tibial portion of the sciatic nerve was evaluated histologically and morphometrically. A clear difference between the distal segments of the healthy contralateral and the repaired tibial portion of the sciatic nerve was detectable, validating the model.

**Conclusion** By transecting the isolated tibial portion of the rabbit sciatic nerve and leaving the peroneal portion intact, it was possible to eliminate automutilation behavior.

## Introduction

Peripheral nerves are particularly susceptible to injury by trauma due to their long course and lack of skeletal protection. These injuries may cause severe distress in the form of motor dysfunction or debilitating neuropathic pain. Peripheral nerve injuries have a significant impact on the quality of life of the patients, as well as health care costs for patients and society. Attempts to correct these potentially life-long disabilities, require numerous reconstructive and rehabilitative sessions, often with dismal recoveries. Less than half of the patients regain significant motor or sensory functions.

While peripheral nerves do have the ability to regenerate spontaneously, the outcome is limited depending on nerve size, distance between the transected ends (nerve gap and defect length), eventual neuroma formation, and scar tissue development, consequently surgical intervention is often required. 1 In instances where the length of the nerve gap does not allow for approximation, or if this will cause excessive traction on the nerve, interposition of autologous nerve grafts is the gold-standard repair approach. 2 Despite the careful selection of a donor nerve, there still exists substantial burden from autologous nerve harvesting which has prompted the medical field to further explore alternatives.

Novel nerve graft developments need to be preclinically evaluated for their potential to substitute autologous nerve grafts. After efforts to limit in vivo animal studies have been exhausted, animal models need to be selected for preclinical evaluation. Beyond the selection of the type of nerve injury, the appropriate animal species and nerve must also be selected to achieve the experimental study goals.

The most commonly studied nerve is the sciatic nerve which innervates the hindlimb. 3 4 While the rat model has been the most widely utilized animal model in peripheral nerve regeneration research, 5 the rabbit model is thought to allow for closer modeling large gap lengths repair approaches.

In the rabbit, the sacral plexus is composed of the 4–7 lumbar nerves and the 1–3 sacral nerves. The main branches of the plexus include the femoral, obturator, and sciatic nerves. The sciatic nerve passes through the sciatic notch and becomes visible near the piriform muscle. It gives off branches that innervate the gluteal, semitendinosus, and semimembranosus muscles. It branches near the knee to form the tibial and peroneal nerve. The tibial nerve innervates the gastrocnemius, soleus, tibialis posterior, flexor digitorum longus, and flexor hallucis longus muscles. In the foot, it innervates the medial and lateral plantar nerves. The peroneal nerve splits to innervate the peroneus longus and peroneus brevis superficially and the deep peroneal nerve innervates the tibialis anterior, extensor hallucis longus, peroneus tertius, and extensor digitorum longus and brevis muscles. 6

We have conducted a review of 40 reports in which a rabbit animal model was used for evaluation of nerve regeneration. 7 8 9 10 11 12 13 14 15 16 17 18 19 20 21 22 23 24 25 26 27 28 29 30 31 32 33 34 35 36 37 38 39 40 41 42 43 44 45 46 To date, the sciatic nerve injury and repair model is the most well-documented nerve injury model in rabbits with 45% of studies performed using the sciatic nerve. In a study by Hsu et al, 19 a 2-cm section of the sciatic nerve was removed after making an incision from the greater trochanter to the mi-calf and splitting the muscle to expose the sciatic nerve. However, the authors note that there is limited data on functional recovery, and the metrics used for rats, such as the sciatic functional index (SFI) and dynamic catwalks (DCW), were not applicable to rabbits. They developed their own metric based on limb extension associated with peak amplitude of compound muscle potential in electrodiagnostical testing. 19 In an effort to better analyze nerve regeneration and provide another model of nerve injury, recent studies have been performed in an attempt to better characterize the rabbit brachial plexus and its motor innervation. 47 However, nerve selection beyond the sciatic nerve or its divisions has been limited, with the peroneal nerve being the second most well-studied nerve in just 18% of studies. An isolated injury to the tibial nerve has been selected in only 8% of studies. 18 48 Hill et al 18 made a longitudinal incision in the hindlimb to expose the tibial nerve. The nerve was then transected 1-cm proximal to its insertion into the gastrocnemius muscle. A 2- or 3-cm segment of the tibial nerve was excised, and the nerve allowed retracting. This study highlights a major issue; despite efforts to prevent autotomy, such as the use of E-collars, 15% of their rabbits had to be eliminated from the study due to excessive autotomy of the foot.

### Autotomy and Mitigation Strategies

The problem of autotomy is not unique to the study by Hill et al. 18 A significant problem with peripheral nerve reconstruction models is the animals' tendency to attack or otherwise damage the denervated digits or limb. 49 Automutilation behavior was first described in rats and mice and referred to as autotomy. 50 Autotomy begins with the nibbling of the toenail and over time this behavior progresses proximally to the phalanges and limb, also infection and edema may follow and forces the subsequent sacrifice of the animal. Events of amputation or sacrifice of the animal make behavioral analysis of functional regeneration impossible.

Automutilation behavior after neurotmesis is also known to occur in rabbits, for example, after sciatic nerve injury. 18 51 52 Our literature review of peripheral nerve rabbit models showed a 30% complication rate with 29% of complications attributed to autotomy. Of interest in the rabbit model are the strain and gender selection. Approximately 65% of cases reported, used the New Zealand Rabbit ( *Oryctolagus cuniculus* ). Studies on this strain are privileged due to good biocompatibility of experimental prosthetic materials. 53 Japanese, European, and Vienna rabbits have also been used at 16, 8, and 3%, respectively. The complication rate appears to be pervasive in male rabbits; with 66% of autotomy cases found occurring in males as opposed to 16% in females, although in some studies either complications were not mentioned or complications were reported regardless gender differences.

Our own attempts with diverse methods applied to prevent autotomy in rats and rabbits did unfortunately not result in the approval of one single or combined method. As utilization of rabbit models for large gap peripheral nerve regeneration studies grows, there continues to be a need to refine the model to alleviate this distress to the rabbit. 54 55

The sciatic nerve divides into the peroneal and tibial nerves. Branches of the peroneal nerve innervate the dorsal and lateral areas of the hindlimb, while branches of the tibial nerve innervate structures of the medial and plantar surface of the hindlimb. In the present study, we describe our attempt to modify the surgical approach by performing an isolated lesion to the tibial portion of the sciatic nerve to preserve sensation to the dorsal aspect of the hindlimb and paw innervated by the peroneal nerve, and thereby to eliminate automutilation behavior.

## Materials and Methods

### Animals

All animal experiments were approved by the Institutional Animal Care and Usage Committee and adhered strictly to the Animal Care guidelines. Twenty female New Zealand White rabbits, weighing 2.5 to 3.0 kg each, were brought to the vivarium 2 weeks before the procedure and housed two per cage in a 12-hour light/dark cycle, with free access to food and water. The animals were marked, and each animal was ascribed to a test group by an independent researcher.

### Experimental Design and Surgical Technique

**Fig. 1:**
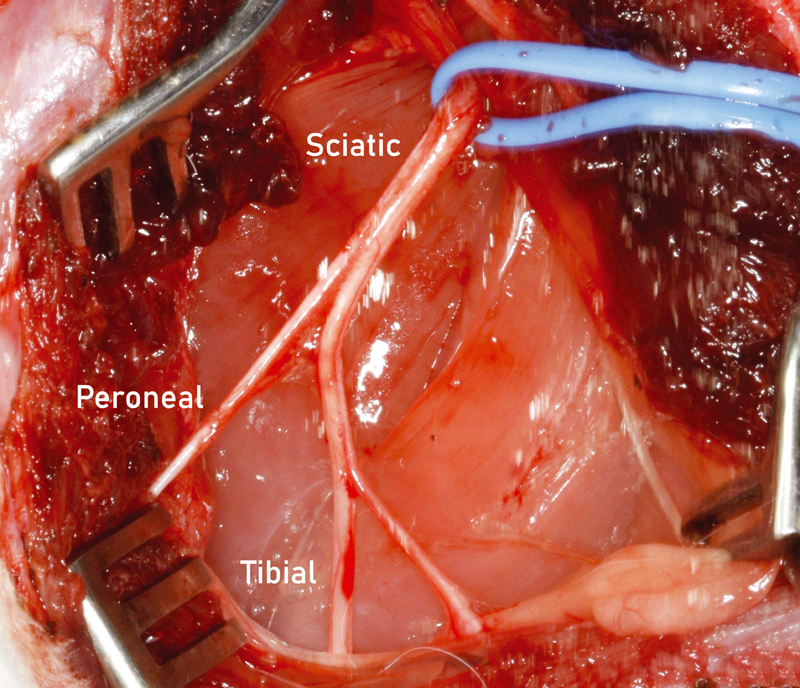
Anatomy of the rabbit sciatic nerve. The sciatic nerve divides into its main branches the peroneal nerve and the tibial nerve. The tibial nerve is the largest branch and gives rise to the sural nerve branch at midthigh level.

General anesthesia was induced with an intraperitoneal injection of medetomidine (0.25 mg/kg) and ketamine (15 mg/kg). The operation on the tibial portion of the sciatic nerve was performed on the left hindlimb. The rabbit was put in a prone position, the hindlimbs abducted and the skin over the lateral and caudal aspect of the limb up to the lumbar midline was sheared. An incision of approximately 6 cm in length was made along the fusion line of the muscles. The fascia was sharply divided and the two muscles (biceps femoris and semimembranosus) were bluntly retracted to enable access to the sciatic, peroneal, and tibial nerves. In one case, the entire sciatic nerve was transected, leading to a severe autotomy of the operated hindlimb; the rabbit was excluded from the study. Using a microscope, the tibial portion of the sciatic nerve was exposed and separated from the peroneal portion ( Fig. 1 ). The tibial portion of the sciatic nerve was transected proximally and distally, and a 2.6-cm gap created between the two ends. Then the rabbits were treated according to their group allocation: (1) group I—autologous nerve graft (control; *n* = 8); (2) group II—Chitosan tube (Reaxon Nerve Guide, Medovent GmbH, Germany; *n* = 12).

**Fig. 2:**
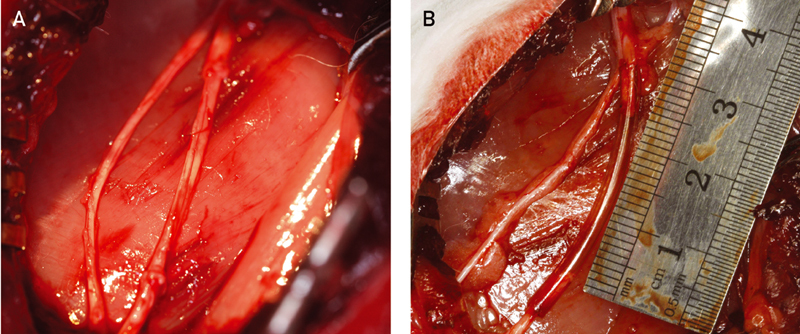
Reconstruction of the tibial portion of the sciatic nerve. The proximal and distal ends of the nerve were either reconstructed with autologous nerve graft (ANG), ( **A** ) or fixed by 10–0 epineurial sutures each with a 2-mm overlap inside 3-cm chitosan tube ( **B** ), creating a 2.6-cm gap between the two ends and microsurgically reconnected.

In group I, the 2.6-cm piece of the tibial nerve was reversed and transplanted. Immediately thereafter, an end-to-end anastomosis was performed between the peripheral nerve segment of 2.6-cm length and the proximal and distal parts of the left tibial nerve, using 10–0 sutures. Coaptation of the nerve fascicles was performed to preserve all the fascicles within the epineurial sac ( Fig. 2A ). In group II, 1-cm nerve segment was removed and a 2.6-cm gap between the two ends created. The proximal and distal ends of the nerve were fixed by 10–0 epineurial sutures each with a 2-mm overlap inside 3-cm Reaxon Nerve Guides (preimmersed in saline), and microsurgically reconnected ( Fig. 2B ). Then the muscles were sutured using 3–0 Vicryl threads and the skin was closed using special metal staples. Postoperative period was 6 months.

### Analysis of Axonal Regeneration—Nerve Stereology and Morphometry

Distal nerve segments of the lesioned left side (three for autograft and *n* = 4 for chitosan tube), as well as healthy control samples ( *n* = 10) of the contralateral right side, were explanted for histomorphometry. Morphometrical analyses were performed according to our previous work. 56 57 58

After fixating distal nerve segments in Karnovsky's solution (2% paraformaldehyde, 2.5% glutaraldehyde in 0.2 M sodium cacodylate buffer, pH = 7.3) for 24 hours, 59 samples were subjected to 0.1-M sodium cacodylate buffer containing 7.5% sucrose. For postfixation, samples were transferred to percentage osmium tetroxide for 1.5 hours. We used 1% potassium dichromate (for 24 hours), 25% ethanol (for 24 hours), and hematoxylin (0.5% in 70% ethanol for 24 hours) for staining of myelin sheaths. Samples were embedded into EPON and afterward cut into semithin cross-sections (1-µm thickness). To additionally enhance staining of the myelin sheaths, sections were stained with toluidine blue. Mowiol (Merck Millipore, Massachusetts, United States) was used to mount the sections.

Evaluation of semithin nerve cross-sections were evaluated as described earlier 56 by using a BX50 microscope (Olympus Europa SE & Co. KG, Hamburg, Germany) which was expanded with a prior controller (MBF Bioscience, Williston, Vermont, United States). For analyses, two sections were randomly selected by using the Stereo Investigator software version 11.04 (MBF Bioscience). A two-dimensional procedure (optical fractionator; grid size: 150 × 150 µm ^2^ ; counting frame size: 30 × 30 µm ^2^ ; counting of “fiber tops” as suggested by Geuna and colleagues 60 ) was used to determine the cross-sectional area (in ×20 magnification), the total number of myelinated fibers (in ×100 magnification), and the nerve fiber density. Samples from the autologous nerve graft repair ( *n* = 3) and healthy control nerve samples ( *n* = 10) were included into stereological and statistical evaluation. Samples from the chitosan tube repair group did not demonstrate axonal profiles in sufficient number for quantitative analysis.

After stereological evaluation, analyses of nerve morphometry were performed by taking photomicrographs of four randomly selected areas of each cross-section (in ×100 magnification). Axon and fiber diameters, myelin thicknesses, and *g* -ratios displayed the parameters which were analyzed. By using the software *g* -ratio plug-in ( *http://gratio.efil.de/* ) in ImageJ version 1.48 (National Institutes of Health, Bethesda, Maryland, United States), 10 axons per picture, leading to 80 axons per animal, were evaluated totally. To be able to calculate axon and fiber diameters, we made the assumption that their shape is circular as described by Geuna and colleagues. 60

## Results

We changed the surgical approach for the rabbit sciatic nerve injury and repair model from lesioning all sciatic nerve (peroneal and tibial portion) to lesioning just the tibial portion ( Fig. 1 ) with the hope that this nerve selection may be one method of reducing or even completely preventing autotomy behavior. By preferentially removing a segment of the tibial portion ( Fig. 2 ), leaving the peroneal nerve intact, sensation at the dorsal aspect of the hindlimb should be preserved. We have not seen a case of autotomy in 20 out of the 20 female rabbits. In one separate single case, due to a transection of the entire sciatic nerve, autophagy was demonstrated within 14 days after surgery and the animal was excluded from the experiment.

As our laboratory furthered our research on nerve reconstruction model, it was necessary to obtain a larger animal model to achieve the desired gap length. The technique of sciatic nerve neurotmesis and reconstruction 61 62 63 that had been used in our rat models was attempted on the rabbit model yielding gross autophagy and early termination of the experiment. In an attempt to avoid further autophagy, the neurotmesis was selectively performed on the tibial portion of the sciatic nerve ( Fig. 2 ). Preservation of the peroneal branch was implemented with the aim that the preserved partial innervation of the limb would limit autophagy. This modified procedure was found to completely eliminate autophagy in the acute peripheral nerve injury of our rabbit model.

**Fig. 3:**
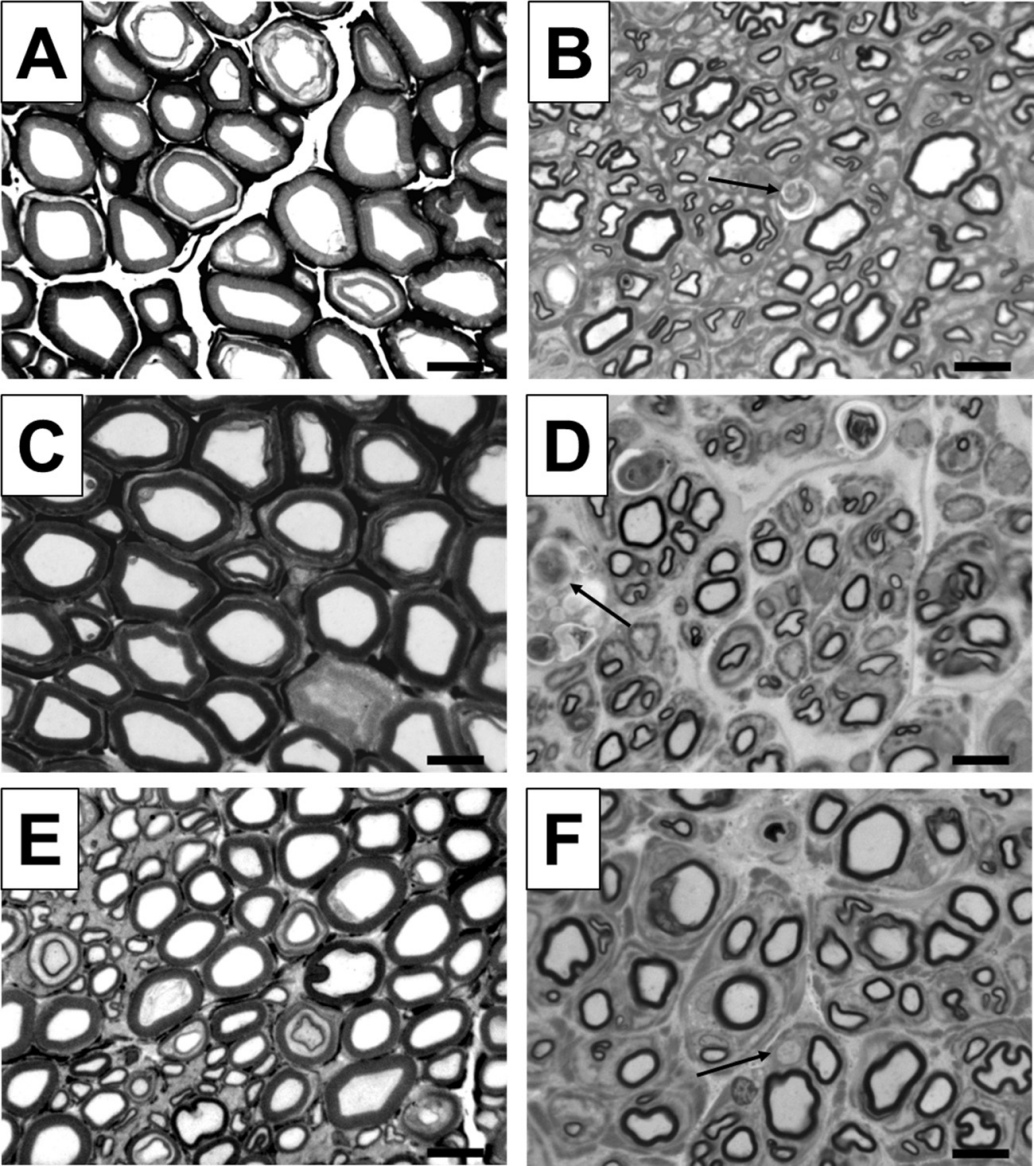
Representative pictures of toluidine blue-stained semi-thin cross-sections of distal nerve segments 6 months after reconstruction surgery. Images show healthy nerve segments ( **A, C, E** ) serving as control compared with distal nerve segments of sciatic nerves, which were reconstructed with autologous nerve grafts (ANGs) ( **B, D, F** ). Healthy nerve samples reveal axons with large diameters and thick myelin sheaths. Pictures of distal nerve segments of autologous nerve graft reconstructed sciatic nerves show successfully regenerated smaller axons with thinner myelin sheaths as well as axonal degeneration (indicated by arrow). Black scale bars display 10 µm.

**Fig. 4:**
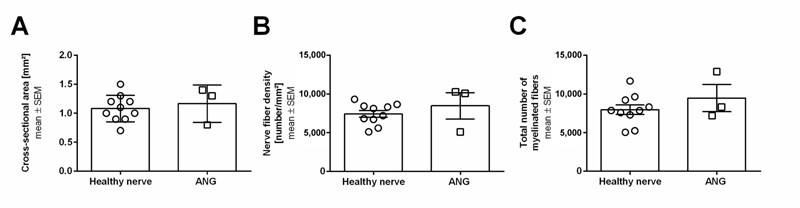
Scatter plot with bar graphs displaying results from histomorphometrical analyses of distal nerve segments from healthy or autologous nerve graft reconstructed sciatic nerves at 6 months of postsurgery. Cross-sectional areas ( **A** ), nerve fiber densities ( **B** ), and total numbers of myelinated fibers ( **C** ) are shown. No significant differences ( *p* < 0.05) were detected by Mann–Whitney test. Results are presented as mean ± SEM (healthy nerve: *n* = 10; ANG: *n* = 3). ANG, autologous nerve graft; SEM, standard error of mean.

**Fig. 5:**
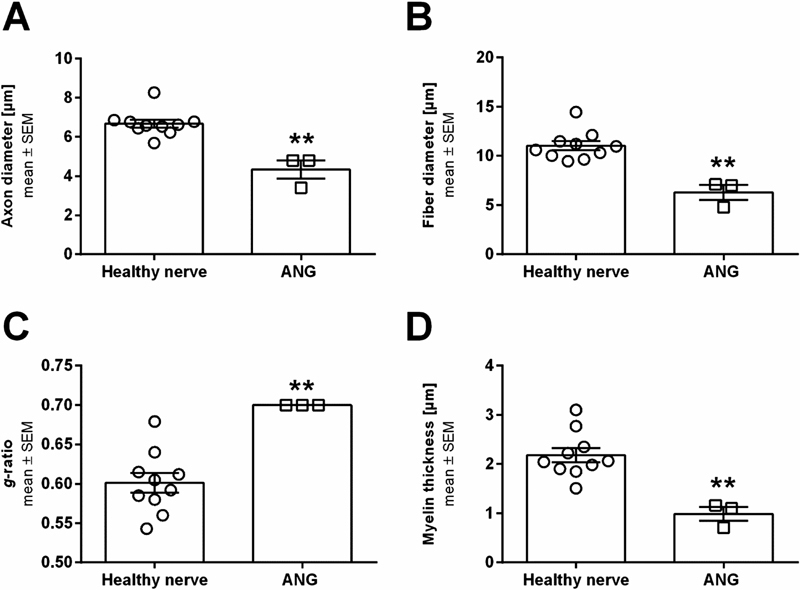
Scatter plot with bar graphs displaying results from histomorphometrical analyses of distal nerve segments from healthy or autologous nerve graft reconstructed sciatic nerves at 6 months post-surgery. Axon diameters ( **A** ), fiber diameters ( **B** ), *g* -ratios ( **C** ), and myelin thicknesses ( **D** ) are shown. Significant differences ( *p* < 0.05) were detected by Mann–Whitney test (** *p* < 0.01 vs. healthy nerve). Results are presented as mean ± SEM (healthy nerve: *n* = 10; ANG: *n* = 3). ANG, autologous nerve graft; SEM, standard error of mean.

With regard to the analysis of axonal regeneration in this modified model, we could clearly detect differences. As shown in Fig. 3 , healthy nerve samples reveal axons with large diameters and thick myelin sheaths, while distal nerve segments of autologous nerve graft reconstructed tibial nerves show successfully regenerated smaller axons with thinner myelin sheaths, as well as axonal degeneration. Quantitative analysis was also possible in the distal nerve segments obtained in our modified model ( Figs. 4 and 5 ). While fiber counts did not demonstrate significant differences, the morphometrical analysis of axonal size parameters revealed the same. In contrast to detection of axonal regeneration, distal nerve segments after repair with chitosan tube appeared significantly degenerated and did not allow quantitative analysis.

## Discussion

The sciatic nerve injury and repair model has been extensively used in peripheral nerve regeneration testing, particularly in rats due to their size, ease of surgical access, presence of sensory and motor fibers, established behavioral functional tests, and number of comparable studies. 4 5 49 64 65 66 67 Because the model has been so well developed, it has extended into larger animal models. However, the use of such a model in larger animals has significant drawbacks.

Our experience with autotomy in rabbits following sciatic neurotmesis has, for this reason, prompted a change in our procedure. We have found that nerve selection may be one method of reducing this autotomy. By preferentially removing a segment of the tibial nerve, leaving the peroneal nerve preserved, sensation to the dorsal aspect of the hindlimb should remain intact. Thus, in our model, we transect only the tibial nerve of rabbits, with the intention of preserving sensation to part of the hindlimb with the aim of limiting or preventing autophagy.

Despite the induced loss of sensation to the plantar surface of the foot, by maintaining dorsal innervation, we have detected a remarkable reduction in autophagy. Of the 20 female rabbits operated, none showed a sign of autophagy. This absence of autophagy is remarkable compared with the 16% found in the sciatic nerve model of female rabbits. We must emphasize that our study was limited to female New Zealand rabbits. To further validate the model, it should be performed on male rabbits to see if the complication rate can be similarly lowered from that of the current 66% or eliminated altogether. 7 8 9 10 11 12 13 14 15 16 17 18 19 20 21 22 23 24 25 26 27 28 29 30 31 32 33 34 35 36 37 38 39 40 41 42 43 44 45 46

## Limitations

One limitation of using a subsidiary of the sciatic nerve is a decreased resectable length. It is not possible to perform a graft greater than 3 cm, as it involves nearly the entire length of the nerve; clinically, peripheral nerve reconstruction is not performed on the entire length of the injured peripheral nerve. Our study utilized a 3-cm nerve conduit, allowing for 2 mm of distal and proximal nerve coverage, providing a 2.6-cm nerve gap, making our modified sciatic nerve model an extreme model. If a larger nerve gap is desired, for example 7 to 8 cm, another animal model, such as a sheep or monkey, may need to be selected.

Unfortunately, in the follow-up period, no functional studies were performed, due to the lack of a functional test that could examine the injured tibial nerve in the rabbit. While this is a drawback in comparison to the sciatic model in rats, the development of relevant functional testing is complicated by vast difference in gait and movement between rats, rabbits, and humans. Thus, very few functional tests have been developed for the sciatic nerve injury and repair model in rabbits and may be a focus of future development. Consistent with all other nerve regeneration models, histological evaluation can still be performed at the end of the follow-up period, and we have demonstrated its value in a peripheral nerve injury model, as well as in our modified sciatic nerve model.

## Conclusion

As our research on nerve graft guides progressed, it was necessary to obtain a larger animal model to achieve the desired gap length. The technique of sciatic nerve neurotmesis that had been used in our rat models 61 62 63 was attempted on the rabbit model yielding gross autophagy and early termination of the experiment. In an attempt to avoid further autophagy, the neurotmesis was selectively performed on the tibial portion of the sciatic nerve with preservation of the peroneal portion. This was performed with the hope that intact innervation to dorsal part of the limb would limit autophagy. It was found to completely eliminate autophagy; while nerve regeneration of the injured nerve still occurs. With such a remarkable reduction in cases of autophagy, it is our recommendation to further test and develop the described modified sciatic nerve injury model as a standard for peripheral nerve injury model in rabbits.
